# Electrical stimulation attenuates morphological alterations and prevents atrophy of the denervated cranial tibial muscle

**DOI:** 10.1590/S1679-45082017AO3808

**Published:** 2017

**Authors:** Cleuber Rodrigo de Souza Bueno, Mizael Pereira, Idvaldo Aparecido Favaretto, Carlos Henrique Fachin Bortoluci, Thais Caroline Pereira dos Santos, Daniel Ventura Dias, Letícia Rossi Daré, Geraldo Marco Rosa

**Affiliations:** 1 Universidade do Sagrado Coração, Bauru, SP, Brazil.; 2 Faculdade de Odontologia, Universidade de São Paulo, Bauru, SP, Brazil.; 3 Universidade Federal do Pampa, Uruguaiana, RS, Brazil.

**Keywords:** Electric stimulation/methods, Muscle, skeletal, Muscular atrophy, Muscle denervation, Rats

## Abstract

**Objective:**

To investigate if electrical stimulation through Russian current is able to maintain morphology of the cranial tibial muscle of experimentally denervated rats.

**Methods:**

Thirty-six Wistar rats were divided into four groups: the Initial Control Group, Final Control Group, Experimental Denervated and Treated Group, Experimental Denervated Group. The electrostimulation was performed with a protocol of Russian current applied three times per week, for 45 days. At the end, the animals were euthanized and histological and morphometric analyses were performed. Data were submitted to statistical analysis with a significance level of p<0.05.

**Results:**

The Experimental Denervated Group and the Experimental Denervated and Treated Group had cross-sectional area of smaller fiber compared to the Final Control Group. However, there was significant difference between the Experimental Denervated Group and Experimental Denervated and Treated Group, showing that electrical stimulation minimized muscle atrophy. The Experimental Denervated and Treated Group and Initial Control Group showed similar results.

**Conclusion:**

Electrical stimulation through Russian current acted favorably in maintaining morphology of the cranial tibial muscle that was experimentally denervated, minimizing muscle atrophy.

## RESUMO

**Objetivo:**

Investigar se a estimulação elétrica pela corrente russa é capaz de manter a morfologia do músculo tibial cranial de ratos desnervados experimentalmente.

**Métodos:**

Foram utilizados 36 ratos Wistar, distribuídos em quatro grupos: Grupo Controle Inicial, Grupo Controle Final, Grupo Experimental Desnervado Tratado, Grupo Experimental Desnervado. A eletroestimulação foi realizada com um protocolo de corrente russa aplicada três vezes por semanas, durante 45 dias. Ao final, os animais foram eutanasiados e, em seguida, foram realizadas as análises histológica e morfométrica. Os dados foram submetidos à análise estatística, com nível de significância de p<0,05.

**Resultados:**

Os Grupos Experimental Desnervado e o Grupo Experimental Desnervado Tratado apresentaram área de secção transversal da fibra menor quando comparados ao Grupo Controle Final. Entretanto, constatou-se diferença significativa entre o Grupo Experimental Desnervado e o Grupo Experimental Desnervado Tratado, mostrando que a estimulação elétrica minimizou atrofia muscular. Ainda, observou-se que o Grupo Experimental Desnervado Tratado apresentou resultados semelhantes ao Grupo Controle Inicial.

**Conclusão:**

A estimulação elétrica por meio da corrente russa foi favorável na manutenção da morfologia do músculo tibial cranial desnervado experimentalmente, minimizando a atrofia muscular.

## INTRODUCTION

Peripheral nerve injuries are common in our society, and they are becoming a public health and social welfare concern. The most common causes of these injuries are gunshot wounds, falls, penetrating injuries, and car accidents.^1-4^

Denervation causes muscle mass loss, a decrease in cross-sectional area of muscle fibers, and a decrease in muscle force production, as well as an increase in perimysial and endomysial connective tissue.^5^This phase is followed by progressive muscle fiber necrosis and apoptosis, with replacement of muscle by fibrous connective tissue and fat.^6-8^

An improved motor recovery after nerve injury and repair can be optimized by accelerating axonal regeneration and slowing progression of atrophy in the target muscle, thus improving the functional capacity of the muscle.^9^

On this regard, several authors suggested a beneficial effect of electrical muscle stimulation after nerve repair during the nerve regeneration process. And also, electrical stimulation is the most direct method suggested for minimizing muscle atrophy during the denervation period.^10-12^

Caierão et al.,^13^ reported a decrease in connective tissue content and an increase in muscle fiber area by using an electrical stimulation protocol in denervated muscle. Matheus et al.,^14^ reported an improvement in the mechanical properties of the muscle.

There is controversy among studies on the therapeutic use of electrical stimulation due to lack of consensus in the use of parameters, intensity, duration, and protocols, requiring further studies to evaluate electrical stimulation protocols, particularly in denervated muscles. However, some studies reported adverse effects of electrical stimulation, which slowed functional recovery and accelerated muscle fiber atrophy in rats after axonotmesis.^15^

Accordingly, we designed this experimental study to evaluate the effects of an electrical stimulation protocol directed to a denervated muscle composed of mixed muscle fibers.

## OBJECTIVE

To investigate if electrical stimulation through Russian current is able to maintain the morphology of the cranial tibial muscle of experimentally denervated rats.

## METHODS

A total of 36 young male Wistar rats (*Rattus norvegiccus*) aged 80 days, weighing on average 250g, were used. The animals were kept in appropriate boxes, with water and feed *ad libitum,* with no movement restriction, on a 12-hour light cycle, at an average temperature of 24° C. The study was approved by the Ethics Committee of the *Universidade do Sagrado Coração* under protocol number 231/10, and conducted in the period of 2011/2012.

The animals were divided into four groups, with nine animals in each group, as follows: Initial Control Group (ICG), whose animals were euthanized at 80 days of life, *i.e.*, in the beginning of the experiment; Final Control Group (FCG), whose animals were euthanized at 125 days of life, *i.e.* at the end of the experiment; Experimental Denervated Group (EDG), whose animals were denervated at 80 days of life, received no treatment, and were euthanized after 45 days; Experimental Denervated and Treated Group (EDTG), whose animals were denervated at 80 days of life, treated post-surgically with electrical stimulation for 45 days, and then euthanized.

### Surgical procedures

The animals were weighed and placed under general anesthesia with an intramuscular injection of tiletamine hydrochloride associated with zolazepan hydrochloride (10mg/kg) in the dorsolateral region of left thigh. The rats were considered anesthetized when the corneal-palpebral reflex was negative, with no motor reaction immediately after painful stimulation of the fat pad of either limb.

The hair of the dorsolateral region of the right hind leg was removed with the animal in prone position on the corkboard, with the limbs fixed with adhesive tape. Antisepsis was performed using povidone iodine with 1% active iodine (Povidine Alcoólico^®^, VIC Pharma Ind. e Com. Ltda., Taquaritinga, SP, Brazil).

An approximately 2cm long longitudinnal incision was made on the leg, in the dorsolateral region of the right thigh. The skin and subcutaneous tissue were detached, and the overlying muscles were divulsed to expose the sciatic nerve, which was completely sectioned. After nerve section, the proximal end was rotated 180° and sutured to the adjacent muscle, and the distal end was rotated 180° and sutured to the subcutaneous tissue, to prevent spontaneous reinnervation.^16^

On the following two days, an analgesic injection of dipyrone, at 150mg/kg (Analgex V^®^, Agener União), was administered intraperitoneally every 12 hours.

### Stimulation protocol

The cranial tibial muscle was chosen for its superficial location, which facilitates the placement of the electrodes for electrical stimulation. A KLD Endophasys R Et 9701 device was used in the study. Skin electrodes, measuring 2cm x 2cm, and an electrode coupling gel were placed on the cranial tibial muscle.

Electrical stimulation was conducted in three weekly sessions, with two application cycles, during a 45 day period. The rats received electrical stimulation applications with Russian current.

The first cycle was conducted for 10 minutes, to stimulate red fibers. For this, a cycle frequency of 2500Hz was used in periods of 0.4ms; the frequency of activation was 30Hz; 3/1 intervals, with 9 seconds of stimulation for each rest period of 27 seconds. The modulation percentage was 50%.

The second cycle was conducted for 10 minutes, to stimulate white fibers. For this, a cycle frequency of 2500Hz was used in periods of 0.4ms; the frequency of activation was 100Hz; 3/1 intervals, with 9 seconds of stimulation for each rest period of 27 seconds. The modulation percentage was 50%.^17^

### Euthanasia and collecting the tibial cranial muscle

The animals were euthanized 45 days after surgery with anesthetic overdose. Afterwards, the right cranial tibial muscle was collected from all animals.

### Histological processing and muscle morphometry

For histological treatment, a fragment of the middle portion of the muscle belly was removed and placed in Tissue-Tek^®^ (O.C.T., Sakura Finetek, Torrance, USA) inclusion media, for freezing in liquid nitrogen, to obtain 10μm histological sections in cryostat (Leica^®^, CM1850, Nussloch, Germany) at -20°C, and prepare histological slides stained with hematoxylin and eosin.^18^

Muscle observation and morphometry were performed using the image capture and analysis software Image-Pro Plus^®^ 6.2 (Media Cybernetics, Bethesda, MD, USA), coupled to an optical microscope (Olympus^®^, BX50, Tokyo, Japan) coupled to a 12 megapixel resolution camera (Olympus^®^ DP-71, Tokyo, Japan). Morphometry was performed with the measurement of 220 fibers per animal, grouped in ascending order of values; the ten largest and the ten smallest muscle fibers were removed.^19^

### Statistical analysis

The Shapiro-Wilk normality test was applied. Subsequently, the groups were compared using one-way variance analysis technique (one-way ANOVA), and Tukey’s tests with significance level p≤0.05 were applied.

## RESULTS

### Macroscopic observations

In all ICG and FCG animals, normal muscle morphology was observed, whereas in the EDG and EDTG groups, no animal showed signs of spontaneous reinnervation resulting from neurotization. Autophagy was observed in the lateral region of the operated limb, in the animals of the EDG group. This occurred due to the section of the sciatic nerve, which indirectly damaged the sural nerve that provides the sensory innervation of the animal limb. Therefore, the animals judged that the limb did not belong to them anymore and practiced self-mutilation. These findings were not observed in the animals of the denervated group treated with the electrical stimulation protocol.

### Histological observations

**Figure 1:**
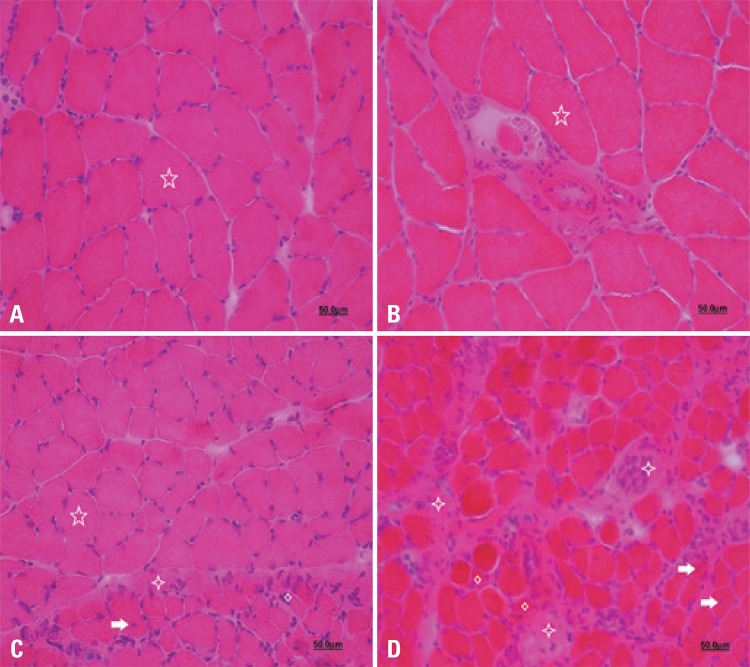
Cross-sectional photomicrograph of the cranial tibial muscle: Initial Control Group (A), Final Control Group (B), Experimental Denervated and Treated Group (C), Experimental Denervated Group (D). See polygonal fibers (five-pointed star), invasion of connective tissue (four-pointed star), central nuclei (white arrow), atrophic muscle fiber (diamond). Staining with hematoxylin and eosin. 40x magnification

The histology of the cross sections showed polygonal muscle fibers in the FCG and ICG groups, with peripheral nuclei, normal fascicular pattern, histological architecture organized with connective tissue, perimysium and endomysial sheaths separating each fascicle and muscle fiber, and demonstrating normal morphology of the skeletal striated muscle tissue. Just one different characteristic was observed among the groups, shown in the morphometric analysis and naturally expected: conspicuously larger muscle fibers in the FCG group, when compared to those in the ICG group, due to the natural and well-known development of rats, which is accelerated when compared to the development of humans in the study period (Figure1).

**Figure 2:**
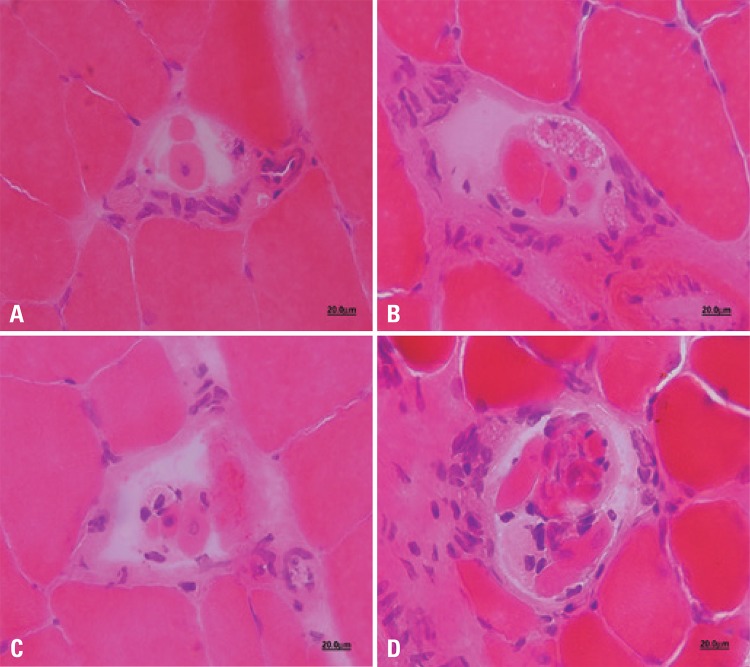
Cross-sectional photomicrograph highlighting muscle spindles: Initial Control Group (A), Final Control Group (B), Experimental Denervated and Treated Group (C), and Experimental Denervated Group (D). See the number and difference of intrafusal fibers in the groups. Staining with hematoxylin and eosin. 100x magnification

On the other hand, EDG presented a great increase in endomysial and perimysial connective tissue, concomitantly with fascicular disorganization, centralized nuclei, small area fibers with angled morphology, characterizing muscular fibers that underwent denervation and are in the process of atrophy (Figure 1). Another finding was the increase in the number of fibers (intrafusal) within the muscle spindle of these animals (Figure 2).

In the EDTG group, characteristics of both FCG and EDG groups were observed. Most of the muscle fibers had peripheral nuclei; central nuclei were also observed, more rarely. Another characteristic was a discrete fascicular disorganization, mainly in the perimysium. Most fibers presented polygonal morphology, with some angled and small area muscle fibers. In addition, no increase in intrafusal fibers was observed, as seen in EDG (Figures 1 and 2).

### Morphometric analysis

**Figure 3:**
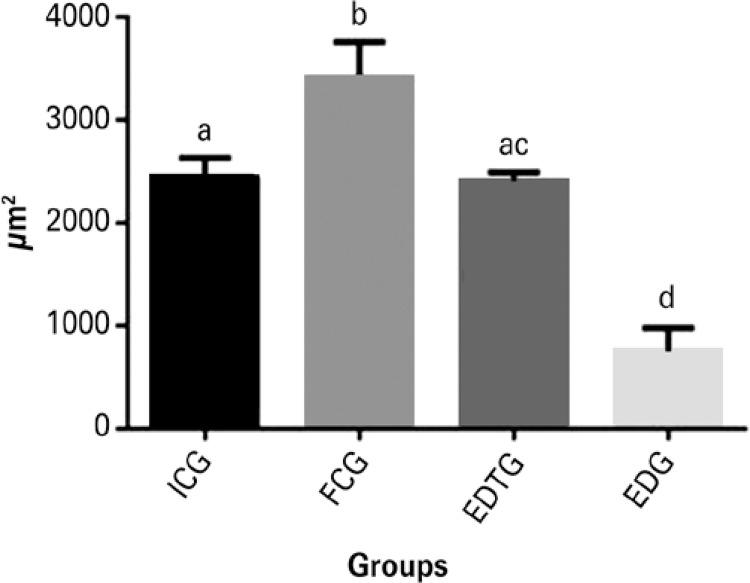
Average and standard deviation of area of muscle fibers in the study groups (different letters represent a statistically significant difference)

ICG: Initial Control Group; FCG: Final Control Group; EDG: Experimental Denervated Group; EDTG: Experimental Denervated and Treated Group.

Morphometric data showed a significant difference between EDTG (2402±94µm^2^) compared to EDG (755±227µm^2^). On the other hand, when comparing ICG (2441±193µm^2^) to EDTG, no difference was observed. When comparing FCG (3414±342µm^2^) to EDTG, FCG had a significantly greater average than EDTG (Figure 3).

## DISCUSSION

In this study, the sciatic nerve surgical neurotmesis (complete section) model was used, and no spontaneous reinnervation was observed in any of the denervated animals (EDG and EDTG). In studies on denervation, special attention is required to the surgical procedure employed.

In lesions classified as neurotmesis, nerve transection is complete, and regeneration necessarily depends on surgical intervention. This is the most adequate model for the study of the denervation process,^13^ contrary to many authors who opt for nerve injury by crushing in their study design. Some authors emphasized that this method may partially preserve the structure of the nerve, which would facilitate regeneration.^20-23^

The morphology reveals the current condition of the muscle. Histological characteristics corresponding to healthy and denervated muscle can be observed. The present histological analysis reveals that electrical stimulation was able to attenuate the morphological changes resulting from denervation.

In this sense, Schmalbruch et al.,^24^ described the presence of central nuclei as a process characteristic of denervation. The EDG showed several central nuclei, unlike the EDTG.

Viguie et al.,^25^ reported that, as the denervation period extends, there is a progressive decrease in the number of myonuclei per muscle fiber, in addition to an initial increase followed by a subsequent drop in the number of satellite cells. These events are associated with a reduction in muscle repair capacity.

Connective tissue hyperplasia is also very evident in denervated muscles, especially in the perimysium and endomysium.^26^ In our study, we found that EDTG minimized the proliferation of connective tissue, unlike EDG, in which a large spacing can be seen histologically between fibers, with interposed connective tissue.

Järvinen et al.,^27^ reported that maintaining a connective tissue balance promotes fiber elongation capacity and traction force, and hence, improves functional capacity.

Guo et al.,^28^ reported that electrical stimulation preserves the satellite cell pool by regularizing its proliferation and apoptosis during the period of disuse-induced atrophy, besides attenuating the loss of myonuclei per fiber, cross-section area and muscular mass. In our study, the histological analysis revealed that electrical stimulation attenuated the morphological changes resulting from denervation.

However, studies on electrical stimulation are still controversial, such as in cases in which it delayed neuromuscular regeneration.^29^ Cavalcante et al.^30^ observed that, when an adequate protocol is applied, electrical stimulation may delay or prevent muscular atrophy. This corroborates what we found in our study, since electrical stimulation prevented muscular atrophy in the EDTG when compared to the EDG.

Several experimental methodologies are used for electrical stimulation. The most commonly used methods are implanted electrodes and skin electrodes with coupling gel. In our study, skin electrodes were used, due to the reduced cost and the need for no other surgical procedure, besides denervation.^30^ We also chose to use skin electrodes in an attempt to bring the study closer to clinical practice, since this is one of the most used and efficient methods.^31^ The application protocol is of extreme relevance for the development and reliability of the experiment results. The protocol of the present study was beneficial, in terms of clinical practice, since it prevented muscular atrophy for 45 days, ensuring a longer period until the muscle is reinnervated. The use of parameters was based on the literature and their clinical applications. The Russian current was chosen because it is one of the most commonly used methods and due to its positive effects on skeletal muscles.^32^

In this sense, the cross-sectional area was studied as an important parameter for the evaluation of skeletal muscles, because it is strongly related to the contraction power of the muscle fiber.

Cavalcante et al.,^30^ concluded that the cross-sectional area should be considered as a parameter for the evaluation of muscle trophism. In our study, we used the cross-sectional area as a parameter and verified that electrical stimulation with the protocol used was able to prevent atrophy of the cranial tibial muscle, when comparing the EDTG with the ICG, showing that electrical stimulation was able to preserve the initial condition of the muscle, as when it suffered the injury. Therefore, electrical stimulation was able to prevent subsequent atrophy. However, when the EDTG was compared with the FCG, electrical stimulation did not promote the natural development of the muscle. A possible explanation for this may be that electrical stimulation does not enable the hypertrophic stimulus needed for the EDTG to achieve results similar to those achieved by the FCG. Similar results were found by Caierão et al.,^13^ who reported that electrical stimulation may not have a hypertrophic stimulus role, or that electrical stimulation was not effective in attenuating the changes in muscle cross-sectional area, since the groups treated with electrical stimulation remained similar to the denervated groups.

Another explanation may be the long period of the experiment. Polônio et al.,^23^ analyzed the effect of electrical stimulation on denervated cranial tibial muscle during various periods, extending the characteristic morphological changes resulting from denervation for up to 28 days, showing that the efficacy of electrical stimulation seems to decrease as the denervation period increases.

In future studies, it would be interesting to perform quantitative tests, using muscle structural proteins, and to study the constitution and types of collagen in muscle envelopes, in addition to associating these with several postoperative periods. Thus, it would be possible to verify possible changes modulated by electrical stimulation at molecular level, mapping its levels of expression in various periods, and adapting the electrical stimulation protocol in the denervated muscle.

## CONCLUSION

Electrical stimulation through Russian current did not provide muscle development in a similar way as it would occur in a healthy muscle, but it was able to prevent atrophy resulting from denervation, and to attenuate the morphological and structural changes that would occur in an untreated denervated muscle.
